# Cell-Selective Altered Cargo Properties of Extracellular Vesicles Following In Vitro Exposures to Intermittent Hypoxia

**DOI:** 10.3390/ijms22115604

**Published:** 2021-05-25

**Authors:** David Sanz-Rubio, Abdelnaby Khalyfa, Zhuanhong Qiao, Jorge Ullate, José M. Marin, Leila Kheirandish-Gozal, David Gozal

**Affiliations:** 1 Department of Child Health, Child Health Research Institute, University of Missouri School of Medicine, Columbia, MO 65201, USA; davidsanzrubio91@gmail.com (D.S.-R.); qiaoz@health.missouri.edu (Z.Q.); jullate92@gmail.com (J.U.); gozall@health.missouri.edu (L.K.-G.); gozald@health.missouri.edu (D.G.); 2 Translational Research Unit, Hospital Universitario Miguel Servet, Instituto de Investigación Sanitaria de Aragón (IISAragón), 50009 Zaragoza, Spain; jmmarint@unizar.es; 3 Centro de Investigación Biomédica en Red de Enfermedades Respiratorias (CIBERes), 28029 Madrid, Spain

**Keywords:** Intermittent hypoxia, extracellular vesicles (EVs), exosomes, OSA, cardiovascular disease, CVD, insulin resistance, adipocytes, macrophages, THP1 cells

## Abstract

Intermittent hypoxia (IH), a hallmark of obstructive sleep apnea (OSA), is associated with cardiovascular and metabolic dysfunction. However, the mechanisms underlying these morbidities remain poorly delineated. Extracellular vesicles (EVs) mediate intercellular communications, play pivotal roles in a multitude of physiological and pathological processes, and could mediate IH-induced cellular effects. Here, the effects of IH on human primary cells and the release of EVs were examined. Microvascular endothelial cells (HMVEC-d), THP1 monocytes, THP1 macrophages M0, THP1 macrophages M1, THP1 macrophages M2, pre-adipocytes, and differentiated adipocytes (HAd) were exposed to either room air (RA) or IH for 24 h. Secreted EVs were isolated and characterized using transmission electron microscopy, nanoparticle tracking analysis, and Western blotting. The effects of each of the cell-derived EVs on endothelial cell (EC) monolayer barrier integrity, on naïve THP1 macrophage polarity, and on adipocyte insulin sensitivity were also evaluated. IH did not alter EVs cell quantal release, but IH-EVs derived from HMVEC-d (*p* < 0.01), THP1 M0 (*p* < 0.01) and HAd (*p* < 0.05) significantly disrupted HMVEC-d monolayer integrity, particularly after H_2_O_2_ pre-conditioning. IH-EVs from HMVEC-d and THP1 M0 elicited M2-polarity changes did not alter insulin sensitivity responses. IH induces cell-selective changes in EVs cargo, which primarily seem to target the emergence of endothelial dysfunction. Thus, changes in EVs cargo from selected cell sources in vivo may play causal roles in some of the adverse outcomes associated with OSA.

## 1. Introduction

Obstructive sleep apnea (OSA) is a highly prevalent disease that is characterized by periodic collapse of the upper airway during sleep, and it is estimated to affect one billion people worldwide [1,2,3]. The repeated episodes of upper airway obstruction during sleep usually result in intermittent hypoxia (IH) and episodic arousals, adversely affecting the quality of sleep, and potentially increasing the risk for both cardiovascular and metabolic dysfunction [2,4,5,6,7,8,9]. Obesity is considered the most important risk factor for OSA, and further promotes the risk of associated morbidities [4,10]. Consequently, and if left untreated, OSA-induced adverse consequences include ischemic heart disease, systemic hypertension, cerebrovascular stroke, atrial fibrillation, diabetes and dyslipidaemias, neurocognitive and mood disorders, excessive daytime sleepiness, increased incidence of motor vehicle accidents, poor quality of life, and increased overall mortality [11,12,13,14,15,16].

In the context of OSA-induced atherosclerosis [16,17,18], endothelial cell dysfunction along with monocyte and macrophage activation play and important role in the initiation and progression of the atheroma plaque [19]. Additionally, sseveral cellular and molecular mechanisms that lead to insulin resistance in the context of OSA have been reported [20,21,22,23,24,25]. To better understand the implications of OSA and its associated morbidities, a substantial effort in the generation of adequate animal models and cell culture systems that reliably mimic the human disease has occurred over the last several decades [9,26,27,28,29], and has led to the proposal of several mechanisms and molecular pathways [23,30,31].

Extracellular vesicles (EVs) are highly heterogeneous nanovesicles that differ in size and biogenesis pathways, and primarily include exosomes (30–150 nm), macrovesicles (100–1000 nm), and apoptotic bodies (800–5000 nm) [32,33,34]. EVs are involved in a large repertoire of organismal processes, particularly regulating cell-to-cell communication and function [35,36,37]. Communication from cell to cell is conveyed by the EVs cargo delivery to target cells via transfer microRNA (miRNA), proteins, RNA, DNA, or lipids [38,39,40], and such cargo is highly dynamic and can be altered in the context of cellular activation, stress, senescence, or hypoxia [41,42,43,44]. Although the specific roles of EVs in the context of OSA subjects have not been fully explored, initial work from our laboratory has shown that circulating EVs from patients with OSA promote endothelial and adipocyte dysfunction and accelerated senescence [9,21,28,37,45,46,47,48].

Intermittent hypoxia, one of the hallmarks of OSA, has now been firmly established as a dominant and major determinant of OSA-related morbidities [18]. Human and animal models of chronic IH seem to show a significant role for IH in the pathogenesis of OSA comorbidity [49,50]. It is being increasingly recognized that IH promotes increased oxidative stress, systemic and vascular inflammation with endothelial dysfunction, increased sympathetic activation, and BP elevations, thus contributing to multiorgan comorbidity [18,51,52,53]. In the present study, we hypothesized that IH exposures would promote changes in the EVs quantity and cargo (miRNA, mRNAs, proteins or lipids) secreted by cells closely related with the pathophysiology of OSA-associated comorbidities. Our aim was to assess the effects of IH on EVs released by endothelial cells, monocytes, naïve and polarized macrophages, pre-adipocytes, and adipocytes, and to explore selective functional aspects on these target cells that promote the occurrence of target organ dysfunction, such as atherosclerosis or insulin resistance. 

## 2. Results

### 2.1. EVs Quantification and Characterization

**Figure 1:**
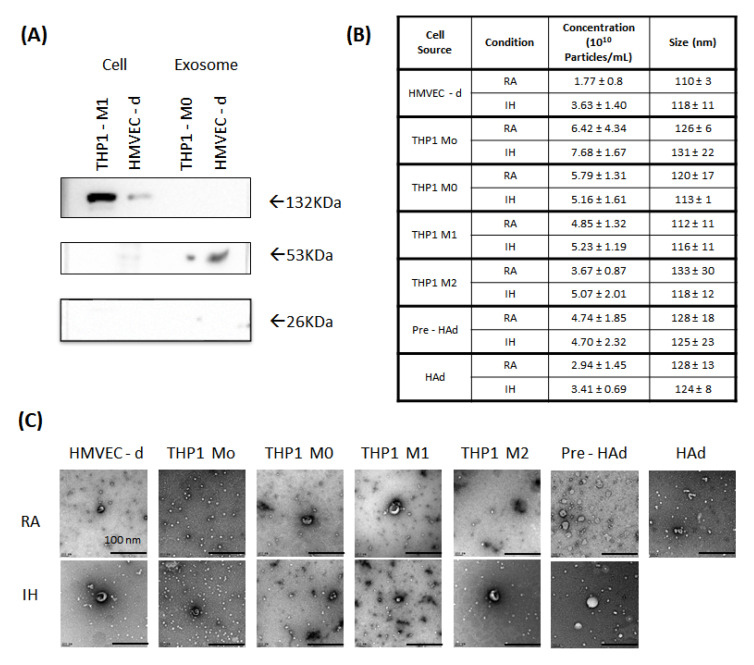
Characterization of extracellular vesicles. (**A**) Western blots of protein markers corresponding to positive markers CD63 (53 KDa) and CD81 (26 KDa) and negative EV marker GM130 (132 kDa). (**B**) Nano tracking analysis (NTA) quantification and median size of the EVs from different cell sources in both conditions room air (RA) and intermittent hypoxia (IH). (**C**) Representative images of electron microscope from the EVs isolated from all cell sources after RA or IH exposure.

EVs derived from different cells lines exposed to IH or RA were characterized using nanoparticle tracking analyzer (NTA), Western blots (WB), and transmission electron microscopy (TEM). Western blot (WB) confirmed the presence of CD63 and CD81 (exosome markers) and the absence of the Golgi apparatus marker GM130 in all EVs isolates (Figure 1A). These CD63 and CD81 proteins are part of tetraspanins super protein family and they are routinely used as positive exosome markers.

The effective size and concentrations range of EVs as determined by NTA and their individual values for each cell source and condition are presented in Figure 1B. Samples of EVs from the different cell sources displayed concentrations with a similar magnitude order, between 10^10^ and 10^11^ nanoparticles/mL. When comparing differences in EVs quantal release between RA and IH conditions, a significant increase in EVs was detected only among HMVEC-d exposed to (*p* = 0.047), with all other cells showing no significant differences. In terms of size distribution, EVs from all cell sources showed a diameter ranging between 35 and 150 nm and peaking between 100 and 135 nm, which is coherent with purified EVs. No differences were observed in EV size in relation to RA or IH exposures. Size and morphology of EVs were evaluated by TEM for all cell sources for both RA and IH conditions. The negative staining of electron microscopy (EM) for each EVs derived from each cell conditions is shown in Figure 1C. As expected from the NTA data, most of the vesicles found were between 30 and 150 nm, and they showed the typical EVs morphology consistent with previously published sources [48,54]. No differences between RA and IH-derived EVs emerged for any of the cells evaluated herein. The only cell source that required a slightly different processing was human adipocyte, for which it was necessary to increase the concentration of the samples from 1:100 to 1:50 to visualize EVs using TEM.

### 2.2. Effects of EVs on Endothelial Cell Monolayer Barrier Integrity

**Figure 2:**
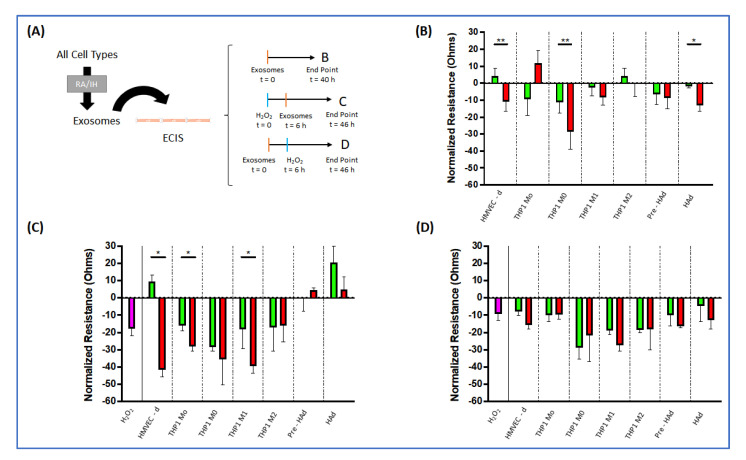
Effect of extracellular vesicles (EVs) on naïve human microvascular endothelial cell (HMVEC - d) tight junction barrier function using ECIS array. (**A**) Graphical scheme of the analyses performed. (**B**) Results from ECIS array after directly exposure to EVs from the different cell sources exposure to both room air (RA, green) and intermittent hypoxia (IH, red). (**C**) Results from ECIS array preconditioned for 6 h with hydrogen peroxide and followed by treating cells with EVs from all cell origins both RA (green) and IH (red). (**D**) Results from ECIS array preconditioned with EVs from all cells sources both RA (green) and IH (red), and addition of hydrogen peroxide after 6 h. * indicates statistical significance, * *p* < 0.05, ** *p* < 0.01.

The effects of EVs from the various cell sources were evaluated under three different conditions. We first evaluated the role of EVs secreted under IH on endothelial dysfunction. This process constitutes an early step in the development of cardiovascular outcomes. The intermediate mechanisms that lead to those consequences are unknown in OSA, and EVs could be crucially involved. Second, we studied the effects of EVs on naïve macrophages; OSA, and more particularly the multiple cycles of IH during sleep, have been broadly associated with systemic low-grade inflammation. EVs could participate in this process through the regulation of both proinflammatory and anti-inflammatory pathways. Third, we also studied the effects of IH derived EVs on insulin sensitivity in naïve adipocytes; In addition to cardiovascular consequences, OSA has been linked with metabolic disorders. EVs could be involved in those metabolic adverse consequences by promoting the insulin resistance under IH conditions. As shown in Figure 2A, we compared the EVs produced by the different cells in RA and IH conditions on a naïve endothelial cell barrier, on an endothelial cell barrier that was pre-treated with H_2_O_2_ for 6 h to promote endothelial cell damage, and on a naïve endothelial cell barrier that was treated with H_2_O_2_ 6 h after treatment with EVs. 

Next, Figure 2B shows the changes induced by EVs (from both RA and IH exposures) to the endothelial monolayer impedance normalized for the concurrent readings when no EVs were added. We observed that the barrier dysfunction induced by EVs from IH conditions was dependent on the cell source. Indeed, EVs secreted from HMVEC-d cells (*p* < 0.01), THP1 M0 (*p* < 0.01) and adipocytes (*p* < 0.05) under IH conditions damaged significantly the monolayer compared to RA, while the EVs derived from the other cell types were void of any effects. The treatment of the monolayer with H_2_O_2_ for 6 h prior to addition of EVs from diverse cell sources altered the monolayer resistance as shown Figure 2C. The first column of Figure 2C presents the damage induced with H_2_O_2_ without any EV treatment. After the pre-treatment with H_2_O_2_, HMVEC-d displayed also higher disruption of the endothelial layer in IH compared to RA (*p* < 0.05). Remarkably, the damage produced by HMVEC-d IH EVs was higher than without pre-treatment. The enhanced susceptibility to the EVs was similar in the EVs secreted by all THP1-derived cells, although in this case both RA and IH showed this increase (*p* < 0.05 in both). Finally, when we inverted the order of addition and treated first the endothelial monolayer with EVs for 6 h, and after that treated with H_2_O_2_, we did not observe any significant change between RA and IH EVs.

### 2.3. Effects of EVs on Naïve Macrophages

**Figure 3:**
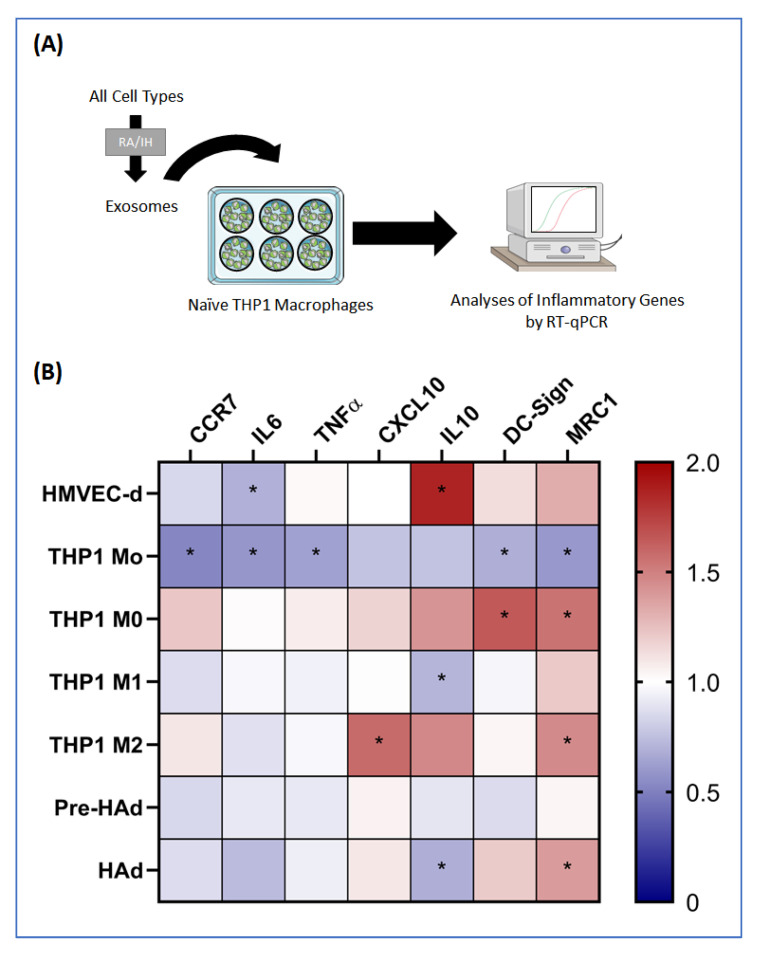
Effect of extracellular vesicles (EVs) on naïve THP1 macrophages. (**A**) Graphical flowchart of the methods used. (**B**) Heat-map corresponding to the fold change between room air and intermittent hypoxia conditions analyzed in four pro-inflammatory genes (CCR7, IL6, TNFα and CXCL10) and three anti-inflammatory genes (IL10, DC-Sign and MRC1). * indicates statistical significance, * *p* < 0.05.

OSA, particularly their continuous cycles of IH during sleep, has been broadly associated with systemic low inflammation. EVs could participate in this process through the regulation of both proinflammatory and anti-inflammatory pathways. In these experiments, we examined the polarity response of naïve THP1 macrophages after 24 of exposure to EVs from the various cell sources after either RA or IH exposures (Figure 3A).

For this purpose, seven genes from macrophages exposed to EVs were evaluated, 4 of these genes were typical of M1 macrophage polarization and 3 were characteristic of M2 macrophage polarity. Figure 3B presents the fold change between IH-derived EVs versus RA-derived EVs for each cell source. IH EVs from HMVEC-d cells and THP1 M0 macrophages promoted M2 polarity in naïve M0 macrophages. Indeed, HMVEC-d IH EVs significantly reduced the pro-inflammatory gene IL6 and increased the anti-inflammatory gene IL10. THP1 M0 IH EVs increased the three anti-inflammatory genes IL10, DC-Sign and MRC-1, showing a significant upregulation of MRC-1 and DC-Sign and a trend towards increased IL10 expression. The rest of the EVs from other cell sources showed divergent effects. IH EVs released by THP1 monocytes decreased tree of the pro-inflammatory genes, CCR7, IL6 and TNFA, but also decreased two of the anti-inflammatory genes, DC-Sign and MRC-1. THP1 M2 IH EVs significantly increased MRC-1 and showed a trend for increasing IL10, but also increased the expression of the pro-inflammatory gene CXCL10. IH EVs secreted by THP1 macrophage M1 and human adipocytes displayed the same profile, showing an increase in one of the anti-inflammatory genes, MRC-1, along with decreases in IL10. Finally, IH EVs from human pre-adipocytes did not show any change in the expression of anti- or pro-inflammatory genes.

**Figure 4:**
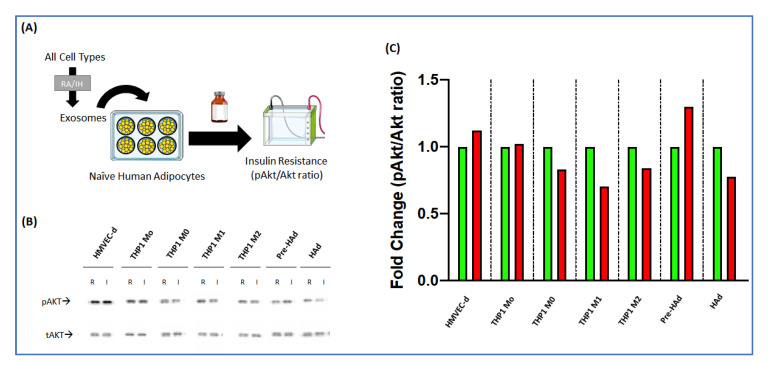
Effect of extracellular vesicles (EVs) on naïve human adipocytes. (**A**) Graphical scheme of the analysis. (**B**) Representative image of bands obtained in the western blot for Akt and pAkt after treatment with EVs from human microvascular endothelial cells (HMVEC-d), THP1 monocytes (THP1 Mo), THP1 macrophages (THP1 M0), THP1 macrophages M1 stimulated (THP1 M1), THP1 macrophages M2 stimulated (THP1 M2), human pre-adipocytes (Pre-HAd) and human adipocytes (HAd) after both room air (R) and intermittent hypoxia (I) exposures. (**C**) Results obtained after quantifying the bands for the ratio pAkt/Akt for EVs from all cell sources after both room air (green) and intermittent hypoxia (red).

Effects of IH derived EVs on insulin sensitivity in naïve adipocytes. In addition to cardiovascular consequences, OSA has been linked with metabolic disorders. EVs could be involved in those mechanisms promoting the insulin resistance under IH conditions. Therefore, we evaluated the changes in insulin induced pAkt/Akt ratio in naïve human adipocytes after 24-h treatment with EVs (Figure 4A). Figure 4C shows the results emanating from such EVs from all the cell sources for both RA and IH conditions. None of the EVs from any of the cells either exposed to IH or RA led to significant changes in insulin-induced pAkt/Akt ratios in naïve adipocytes.

## 3. Discussion

The present study illustrates for the first time the specific and divergent effects of EVs-derived from different cell sources (human endothelial cells, adipocytes, monocytes and macrophages) following IH exposures, and their potential contributions to the adverse consequences commonly associated with OSA (i.e., vascular dysfunction, macrophage polarity changes, and insulin resistance). For example, EVs-derived from HMVEC-d, THP1 macrophages M0 and human adipocytes exposed to IH disrupt the naïve endothelial barrier. Contrary to our initial assumptions, EVs secreted by HMVEC-d and THP1 macrophages M0 exposed to IH promoted an anti-inflammatory profile, with other cells inducing a mixed effect. Furthermore, the ratio of pAKT/tAKT as indicator of insulin sensitivity remained unaltered after exposure to any of the EVs, irrespective of whether the cells had been exposed to IH or RA. Such findings, which are not aligned with the fact that circulating EVs in patients with OSA induce insulin resistance [21,55], suggest that other cells (for example, platelets, lymphocytes, etc.) and possibly other factors that are present in OSA but not reflected in the in vitro IH exposures (e.g., sleep fragmentation) may be operationally involved. However, notwithstanding the dissimilarities between in vitro and in vivo EVs in the context of IH, the current experiments illustrate the potential contributory roles of EVs in OSA-induced morbidities. Our findings support the processes that occur in patients with OSA and their increased risk of developing endothelial dysfunction, atherosclerosis, and definitely other cardiovascular outcomes.

OSA is recognized as an important and independent risk factor for hypertension, coronary heart disease, stroke, mild cognitive dysfunction, and metabolic dysfunction [56,57]. The molecular pathways underlying the deleterious consequences of OSA are under investigations, however, the cellular and the molecular mechanisms remain poorly understood [51,58]. Some clinical and animal studies have demonstrated that endothelial dysfunction is a precursor of atherosclerosis, which is a pathological condition underlying cardiovascular diseases [59]. Of the several mechanisms activated by IH, oxidative stress and activation of inflammatory pathways have emerged as consistent players in endothelial dysfunction in both human and animal studies, as well as in vitro cellular experiments [25,37,60,61,62,63,64,65,66,67,68,69,70,71]. The common mechanisms linking IH, OSA and CVD are complex and likely mediated by multiple mechanisms. For example, human and animal studies have demonstrated that the pathophysiologic changes brought on by OSA, including changes in intrathoracic pressures, hypoxia, and hypercapnia, which may cause structural and electrical changes that predispose to arrhythmia [72]. Additionally, hypoxia and hypercapnia associated with sleep apnea affect sympathetic nerve activity and cause vasoconstriction, and as a result hypertension may ensue, which is a known risk factor for atrial fibrillation [73,74]. Recently, mice exposed to CIH exhibit changes in the passive stiffness of the cardiac tissue extracellular matrix (ECM), a critical factor underlying conduction changes and predisposing to atrial fibrillation [75]. Furthermore, it has been shown that IH can contribute to a vasoconstrictive profile and to pathway-selective vascular IR through caveolin-1 overexpression, and its effect on nitric oxide metabolism [25]. Another mechanism of promotion of endothelial damage associated with IH is the increase in monocytic CCR5 gene expression and the enhanced RANTES-mediated chemotaxis and adhesion [67]). In addition, the serum of OSA patients displayed an inflammatory bioactivity after incubation with endothelial cells [70]. Those mechanisms are not only focused on endothelial cells, but also on other cell types in adipose tissue, the latter potentially playing a crucial role in the negative effects mediated by IH [71]. The cumulative findings from such studies have clearly identified that duration and severity of hypoxia are of great importance, that a multitude of pathways may be operationally recruited, and that interactions between different cells are also major determinants of the overall outcomes. Our data suggest that, at least in vitro, intermittent hypoxia, one of the main consequences of apneic events, is capable of inducing changes in EVs that would promote cardiovascular damage. Understanding of the CVD mechanisms in the context of OSA may allow for more direct targeting of specific pathophysiological contributors. Furthermore, new insights into the molecular pathophysiology of CVD open new opportunities in risk assessment and monitoring of therapeutic responses.

The opportunity to study human cells in culture can increase our insights into healthy and diseased states of the human body [76]. Significant efforts were made to accomplish relevant models of IH in cell cultures, and in parallel we have established a standard protocol for EVs isolation from different cell lines exposed to IH [28,29,54]. Several studies have reported about the release and the biogenesis of EVs in vitro cell cultures [77,78,79,80]. Thus, EVs are crucial in cell-to-cell communication, transferring the cells’ phenotype from the origin cells to the receptor cells [81,82]. White adipose tissues-derived EVs showed immunomodulatory properties which convert macrophages towards a pro-inflammatory phenotype in vitro, and modulate insulin signaling in muscle and liver cells [79,83], 3T3-L1 cell adipocytes [84], and a human preadipocyte differentiated cell model [84]. In our study, we are able to demonstrate that IH can partly reproduce intermediate mechanisms that in real life would be induced by IH in patients with OSA.

A number of studies have highlighted the potential contributions of EVs to human diseases, and OSA is not an exception. Studies on the involvement of EVs in the emergence of endothelial cell dysfunction and adipocyte insulin resistance in both patients suffering from OSA and animal models of OSA have provided robust initial evidence supporting such critical roles [9,21,28,55,64,85,86,87,88,89,90,91,92,93,94]. EV cargo is altered in response to hypoxia. For example, breast cancer cells released exosomes with high levels of miR-210 among their cargo under hypoxic conditions and hypoxic lung cancer cells showed increased levels of miR-23a [43,95]. Furthermore, pancreatic cancer cells increased the releasing of EV in hypoxia although decrease their size and hypoxia-resistant multiple myeloma cells produced higher concentration of exosomes compared to normoxic cells [81,95,96]. In the present study, although a slight increase of release in exosomes occurred after intermittent hypoxia exposure for most cell types, those changes did not reach statistical significance. This fact could be due to the intermittent nature of the hypoxia in our experiments. We therefore assume that most of the functional changes described as response to hypoxia in general, and particularly following IH, are caused by cargo alterations of the EVs, although further research is required in this field.

Endothelial cells play a key role in maintaining constant vascular tension, and vascular endothelial dysfunction, which is characterized by imbalanced vasoconstrictive and vasodilatory molecules, is the earliest sign of a vascular lesion preceding the occurrence of clinically obvious cardiovascular complications in OSA [17,19,97,98]. Hypoxia can also trigger complex intercellular communication, may aggravate endothelial dysfunction and destruction in early pre-atherosclerosis, and induce vascularization in cancer tissue and a progression of the disease [99,100,101]. In addition, hypoxic preconditioning for 24 h of mesenchymal stem cells improves their efficacy in the treatment of cardiovascular diseases [102]. It has been indicated that circulating EVs are in direct constant contact with endothelial cells and regulate endothelial cell proliferation, apoptosis, and migration, thus regulating vascular function [103]. Adipocyte EVs seems to play an important role in endothelium homeostasis, promoting under hypoxic conditions the leukocyte attachment via increasing the levels of PPARγ among their cargo [104]. In addition, previously, we showed that EVs from OSA patient plasma impair endothelial adhesiveness and permeability [9,45,48] which may directly or indirectly trigger or exacerbate cellular endothelium, possibly via oxidative stress-related pathways. Our results suggest that endothelial dysfunction promoted by EVs as response to IH is not only stimulated by endothelial cells, but also by others such as macrophages or adipocytes.

Signaling and communication between endothelial cells and monocytes/macrophages play a critical role in cardiovascular homeostasis and the pathogenesis of atherosclerosis [105]. Macrophages have been categorized into two artificially distinct activation states designated as classical (M1) and alternative (M2), even though there is a much wider spectrum of combinations of the two putatively distinct macrophage phenotypes [106]. In addition to adipocyte-derived factors, an increased release of tumor necrosis factor-α (TNF-α), interleukin-6 (IL-6), monocyte chemoattractant protein-1 (MCP-1), and additional products of macrophages and other cells that populate adipose tissue also play a role in the development of cardiovascular and metabolic risk including insulin resistance [107]. It has been described that under hypoxic conditions EVs produced by mesenchymal stem cells induced a shift toward a M2 differentiation in naïve macrophages [108]. In a similar trend, our study showed that EVs released by endothelial cells and macrophages during IH promote an anti-inflammatory status in naïve macrophages. Furthermore, the secretion of exosomes from adipose tissue macrophages regulates metabolic and inflammatory interactions between adipocytes and macrophages [109]. More recently, injection of secreted exosomes from M2 macrophages into obese mice improved insulin-glucose homeostasis without affecting adiposity [24].

**Figure 5:**
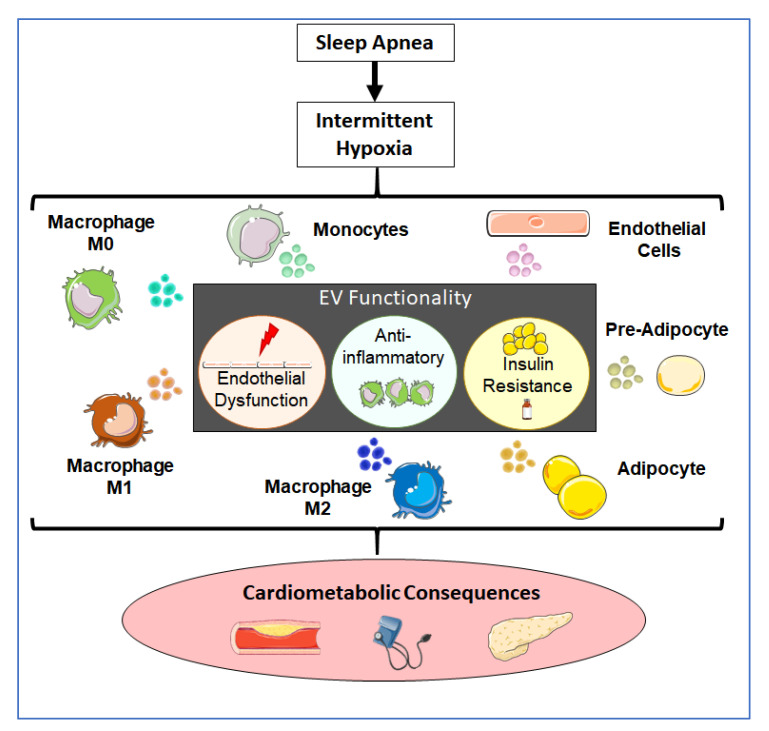
Schema illustrating the hypothetical role of EVs in OSA cardiometabolic consequences. Intermittent hypoxia as a resulting of OSA may alter the EVs cargo produced by different cell types as endothelial cells, macrophages, or adipocytes, modifying the communication between those cells, and promoting the cardiovascular comorbidities commonly associated with OSA and cardiometabolic consequences [21,46,47,60,109].

Recently, we and others showed that EVs play a role in the development of the metabolic dysfunction by serving as a mode of intercellular communication among adipose tissue, liver, skeletal muscle, and immune cells [21,54,79,83,85]. In addition, EVs may play a role in the regulation of peripheral insulin sensitivity, a major component of the pathogenesis of multiple diseases by regulate insulin sensitivity through at least two different mechanisms, i.e., by modulating inflammation or by direct interaction with insulin-responsive organs [110]. In addition, hypoxia has been shown to promote changes in naïve adipocytes in their EVs that induce a reduction in the insulin-stimulated glucose uptake [111]. Our results show that the EVs produced under IH conditions by the different cell types do not modify the insulin resistance in naïve adipocytes, therefore, this effect described in association with OSA could be produced by other mechanisms rather than by the simple interactions of cells with IH-induced EVs cargo changes. We summarized the effects of IH on different cell sources and those of EVs and cardiovascular disease (Figure 5).

This study has several limitations. First, we used in vitro models, a relatively simple and minimalistic approach to evaluate the complex processes that occur in the organism. Our cell cultures were exposed individually to IH, without any interaction with another cell type. Although these experiments are required to examine the specific effects of EVs from each cell source, further research using co-cultures of different cell types in both 2D and 3D would be beneficial [112]. Second, the effects of IH in a cell culture system might be different from the IH that characterizes OSA patients, particularly the number of cycles of IH exposure. Indeed, we only exposed the cells for 24 h as compared to many months and years of IH exposure that occur in OSA patients during their sleep. In addition, other pathological process such as arousal and activation of sympathetic nervous system are acting in those patients and such events cannot be recapitulated in vitro. Finally, in this study we performed a limited number of functional assays. Specially, we only evaluated a specific set of inflammatory genes to evaluate the capacity of EVs to change the inflammatory status and we measured Akt/pAkt ratio changes to evaluate insulin resistance and the effects of EVs in promotion of metabolic dysfunction.

Finally, our results indicate the relevance of IH as an intermediate mechanism for the development of vascular involvement in OSA. The clinical implications of these findings include the need of an effective treatment of OSA. This should include the elimination of nocturnal hypoxemia. It is therefore imperative that the treatment (e.g., with continuous positive airway pressure) be effective. That is to say, there should not be any residual nocturnal hypoxemia in the context of good adherence. On the other hand, given the heterogeneity of OSA and its different degrees of IH severity, the effect of different hypoxemia-degrees and associated secreted EVs at the cellular level should be evaluated in future studies. This would allow for establishing risk stratification concerning hypoxemia data in the management of OSA patients

Exosomes play critical and important roles in the regulation of physiological and pathophysiological processes, recognition and diagnosis, and treatment of CVD [113]. Since exosomes are released by most cells and they are relatively stable in the blood circulation and biological fluids, they have a potential future for preventive and diagnostic applications, and can help to develop future interventions and clinical applications. Furthermore, exploring different cell sources for therapeutic EVs is of utmost interest, because the lipid and surface protein composition of exosomes may be crucial to their function, and preservation of these characteristics is very important [114]. Multiple efforts have focused on the development of diagnostic and therapeutic applications that are based on exosome cargo, primarily RNA encapsulated in exosomes or carried in other carrier subtypes [115]. These EVs are promising nanocarriers for clinical use. However, the clinical applications of EVs are still in a very early stage, and further investigations are required.

In summary, we have shown that in isolated cell culture conditions, specific cell types exposed to IH exhibit divergent changes in the EVs cargo they release and also differ in the functional properties in of their EVs cargo when such cargo is delivered to naïve cells. Intercellular signalling by EVs is a route of cell-cell crosstalk that allows cells to selectively deliver biological messages to specific recipient cells. EVs convey these messages through their distinct cargos consisting of cytokines, proteins, nucleic acids, and lipids, which they transport from the donor cell to the recipient cell. In cardiovascular disease, endothelial- and immune cell-derived EVs are emerging as key players in different stages of disease development. Endothelial cell dysfunction is a key element in the complex pathophysiology of atherogenesis and triggers the release of endothelial cell-derived EVs. However, the effects of EVs in CVD are extremely complex and depend on the cellular origin, the functional state of the releasing cells, the biological content, and the diverse recipient cells. In addition, EVs can mediate the cross-talk between adipose tissues and macrophages that facilitates the deregulation of immune and metabolic homeostasis, raising the potential opportunity for EVs-based therapeutics in obesity, diabetes, or in OSA-induced cardiometabolic disease. Our future work will focus on profiling the genomic, transcriptomic, proteomic, and lipidomic cargo of EVs derived from different endothelial cells and their interactions with other relevant cells under intermittent hypoxic conditions. Such information should help identify hypoxia-specific molecules contributing to end-organ damage in the context of OSA.

## 4. Materials and Methods

### 4.1. Human Endothelial Cells

Human microvascular endothelial cells- dermal (HMVEC-d) were purchased from Lonza (catalog # CC-2543; Lonza, Alpharetta, GA, USA). Cells were grown in endothelial growth medium (EGM-2-MV; Alpharetta, GA, USA) supplemented with 5% fetal bovine serum, FBS, (Life Technologies, Grand Island, NY, USA), and further incubated at 37 °C% CO_2_ in cell culture incubator. The cells were trypsinized and centrifuged at 250× *g* for 5 min, diluted, and re-plated at appropriate densities. All cells were used before passage 4.

### 4.2. Human Monocytes (THP-1)

THP-1 cells, a human monocyte cell line, were purchased from American Type Culture Collection (ATCC TIB-202; ATCC, Baltimore, MD, USA). Cells were cultured in RPMI 1640 (Life Technologies, Grand Island, NY, USA) supplemented with 10% FBS. All THP-1 derived cells were growth at 37 °C and 5% CO_2_. THP-1 derived macrophages (M0) were obtained through differentiation with PMA (phorbol 12-myristate 13-acetate (Sigma-Aldrich, St. Louis, MO, USA) at a concentration of 10 ng/mL in growth medium for 3 days. THP-1 cells were seeded in 6-well plates with a density of 1,500,000 cells per well. Polarization to M1 macrophages was carried out with IFN-γ and LPS at 25 ng/mL and 0.1 ng/mL respectively for 3 days. M2 polarized macrophages were obtained after 3 days of treatment with IL-4 and IL-10 at 25 ng/mL both.

### 4.3. Human Adipocytes

Human adipocytes (adipose derived stem cells, ADSCs) were purchased from Lonza (# PT-5006, Lonza, Alpharetta, GA, USA) and cultured at 37 °C and 5% CO_2_ in pre-adipocyte basal media (PGM-2; Lonza, Alpharetta, GA, USA) supplemented with 10% FBS (Life Technologies, USA). Cells were seeded in 6-well plates with 350,000 cells O_2_ per well in basal medium PGM-2, and after 24 h, cells were differentiated by changing to Bulletkit PGM-2 (PT-9502 & PT-8202; Lonza, USA) media containing 10% FBS, 1 μg/mL insulin, 1 μM dexamethasone (DEX), and 0.5 mM 3-isobutyl-1-methylxanthine. The medium was changed every 2 days for 12 days.

### 4.4. Intermittent Hypoxia (IH) Exposures

Exposures to normoxia and IH were carried out in 6-well plates for all cells. IH (5% O_2_, 5% CO_2_, and balance N_2_ for 30 min alternating with 30-min normoxia (21% O_2_, 5% CO_2_, and balance N_2_), using a custom-designed, computer-controlled incubation chamber attached to an external O_2_-CO_2_ computer-driven controller (Biospherix, Redfield, NY, USA) as previously described [116,117].

HMVEC-d and human pre-adipocytes were seeded with a density of 350,000 cells per well, and incubated in corresponding growth medium for 24 h until confluence. Then, cells were washed with non FBS medium and growth medium was replaced with the equivalent EGM-2 and PBM-2 supplemented with 5% and 10% depleted FBS (centrifuged overnight at 100,000× *g*) respectively. Human adipocytes obtained after differentiation were washed with non FBS medium and differentiation medium was substituted by PBM-2 supplemented with 10% depleted FBS. THP1 monocytes were centrifuged at 250× *g* for 5 min, washed with non FBS RPMI 1640 medium and centrifuged again. After that, they were plated at 1,500,000 cells per well in 10% depleted FBS RPMI 1640 medium and incubated for 2 h before starting the exposure. THP1 naïve macrophages (M0), M1 THP1 macrophages and M2 THP1 macrophages were washed once with non FBS RPMI 1640 medium and RPMI 1640 supplemented with 10% depleted FBS was added.

### 4.5. Isolation of Extracellular Vesicles (EVs)

EVs were isolated from the supernatants using the Total Exosome Isolation kit (Life Technologies, USA) per manufacturer’s guidelines. All experiments were performed with EVs at a 1:100 dilution. Cell supernatants of the different cell types were centrifuged at 3000× *g* and 4 °C for 30 min. Pellets were discarded and for each 1 mL of supernatant, 0.5 mL of precipitation buffer was added. The mix was vortexed and incubated overnight at 4 °C. The solution was then centrifuged at 10,000× *g* and 4°C for 1 h and 15 min and pellets were resuspended in fresh filtered (0.22 µm) Dulbecco’s PBS (DPBS; Life Technologies, Grand Island, NY, USA). EVs stocks were stored at −20 °C.

### 4.6. Nano Tracking Analysis

Nanoparticle concentration and size distribution were determined using a Nanosight NS300 instrument (Malvern Instruments, Worcestershire, UK). All samples were diluted in PBS with 5 mM EDTA to a final volume of 1 mL. Dilution of EVs was adjusted based on the ideal number of particles per frame (20–100 particles/frame) by pretesting the samples. Camera levels were adjusted until all particles were distinctly visible and not exceeding particle signal saturation over 20% (level 13) according to the manufacturer’s software manual (NanoSight NS300 User Manual, MAN0541-01-EN-00, 2017). For each measurement, five 1-min videos were captured at a temperature of 25 °C. After capture, videos were analyzed using NanoSight Software NTA 3.1.

### 4.7. Transmission Electron Microscopy

Isolated EVs solutions were diluted 1:100 in Dulbecco’s PBS (DPBS; Life Technologies, Grand Island, NY, USA), and 5 μL of diluted exosomes were placed on parafilm before the Formvar/Carbon-coated grid are placed on top of exosome drops and allowed to stand for 2 min. Grids with adherent exosomes were washed three times with 30 μL DPBS drops and fixed with 2% paraformaldehyde in DPBS for 5 min. Finally, grids were incubated with 30 μL drops of 2% uranyl acetate and examined by electron microscopy [54]. The samples were washed with distilled water seven times (2 min each), and then they were viewed under a FEI Tecnai F30 Twin (Atlanta, GA, USA) transmission EM to measure the size of the isolated EVs [54].

### 4.8. Electric Cell-Substrate Impedance Sensing (ECIS) Endothelial Cell Assay

Endothelial cell monolayer barrier integrity was measured using the ECIS system (Applied Biophysics, Troy, NY, USA) which monitors the electrical impedance across small 250-micrometer diameter electrodes within wells used as substrates for cell growth. Baseline measurements were established for each array using culture medium (300 μL/well). HMVEC-d cells were seeded (70 × 105 cells/well) onto an 8W10E array and grown to confluence with media containing 5% depleted FBS. Once confluent, equivalent amounts of EVs were added into the wells, and trans-endothelial electrical resistance was monitored continuously for up to 40 h.

### 4.9. Effect of EVs on Macrophage Polarity

THP-1 monocytes were differentiated for 3 days as previously described in a 6-well plate with a density of 1,500,000 cells per well. Then, the media was replaced with RPMI 1640 supplemented with depleted FBS and EVs from the different cell sources were added to the naïve macrophages for 24 h. Supernatants were removed, and cells were collected with Qiazol (Qiagen, Germantown, MD, USA). Total RNA was isolated from THP-1 macrophages and prepared using RNeasy Lipid Tissue Mini Kit (Qiagen, Germantown, MD, USA) as described by the manufacturer’s protocol. RT-qPCR analysis was performed for selected mRNAs using QuantStudio 3 Real-Time PCR System (Thermo Fisher Scientific, Hanover Park, IL, USA).

### 4.10. Effect of EVs on Insulin Sensitivity in Naïve Adipocytes

Human pre-adipocytes were grown in 12-well plates, and cells were differentiated as described above. By day 14 of differentiation, media were replaced with growth media containing 10% depleted FBS. Exosomes were added for 24 h and adipocytes were treated with 5 nm insulin (Sigma-Aldrich, USA) at 37 °C for 30 min. Adipocytes were rinsed with cold PBS and 2% SDS (200 μL). Protein concentrations of the cell lysates were determined using the BCA Kit (Life Technologies, Grand Island, NY, USA). The lysates were separated on Novex WedgeWell 12% Tris-Glycine gels (Life Technologies, Grand Island, NY, USA) and transferred to nitrocellulose membranes, incubated in blocking buffer (5% non-fat dry milk in TBST) followed by phosphoAkt (Ser473) antibody (Cell Signaling Technology, Danvers, MA, USA) or total Akt antibody (Cell Signalling Technology, US) overnight at 4 °C. Immunoreactive bands were visualized using an enhanced and quantified by the Image Lab software (Chemidoc XRS+; Bio-Rad, Hercules, CA, USA).

### 4.11. Statistical Analysis

Data are reported as mean ± standard deviation (S.D.) unless otherwise indicated. Data presented are from at least 3–4 independent experiments. Statistical analyses were performed using GraphPad Prism V.8. Comparisons between groups were performed using unpaired Student *t*-tests. A two-tailed *P* value < 0.05 was defined as achieving statistical significance.

## Data Availability

Data available on request.
